# Hydroxychloroquine Inhibits Macrophage Activation and Attenuates Renal Fibrosis After Ischemia-Reperfusion Injury

**DOI:** 10.3389/fimmu.2021.645100

**Published:** 2021-04-14

**Authors:** Haofeng Zheng, Yannan Zhang, Jiannan He, Zhe Yang, Rui Zhang, Lei Li, Zihuan Luo, Yongrong Ye, Qiquan Sun

**Affiliations:** Organ Transplantation Research Institute of Sun Yat-sen University, The Third Affiliated Hospital of Sun Yat-sen University, Guangzhou, China

**Keywords:** hydroxychloroquine, chronic kidney disease, fibrosis, macrophage, inflammation

## Abstract

Chronic kidney disease (CKD), which is associated with high morbidity, remains a worldwide health concern, while effective therapies remain limited. Hydroxychloroquine (HCQ), which mainly targets toll-like receptor-7 (TLR-7) and TLR-9, is associated with a lower risk of incident CKD. Taking into account that TLR-9 is involved in the development of renal fibrosis and serves as a potential therapy target for CKD, we investigated whether HCQ could attenuate CKD *via* TLR-9 signal pathway. The effects of HCQ on renal tubulointerstitial fibrosis were further explored using a mouse model of renal tubulointerstitial fibrosis after ischemia/reperfusion injury. Bone marrow-derived macrophages were isolated to explore the effects of HCQ *in vitro*. Judicious use of HCQ efficiently inhibited the activation of macrophages and MAPK signaling pathways, thereby attenuating renal fibrosis *in vivo*. In an *in vitro* model, results showed that HCQ promoted apoptosis of macrophages and inhibited activation of macrophages, especially M2 macrophages, in a dose-dependent manner. Because TLR-7 is not involved in the development of CKD post-injury, a TLR-9 knockout mouse was used to explore the mechanisms of HCQ. The effects of HCQ on renal fibrosis and macrophages decreased after depletion of TLR-9 *in vivo* and *in vitro*. Taken together, this study indicated that proper use of HCQ could be a new strategy for anti-fibrotic therapy and that TLR-9 could be a potential therapeutic target for CKD following acute kidney injury.

## Introduction

Acute kidney injury (AKI) is a worldwide health problem, and approximately two million deaths occur due to AKI. The incidence of AKI is rising, and patients with severe AKI have a mortality rate of >50% (1, 2). AKI is considered to represent not only acute injury, but also influences recovery from injury. Approximately 13% hospitalized patients experience AKI annually in the US; moreover, around 10% individuals worldwide develop chronic kidney disease (CKD) and end-stage renal disease consequent to AKI (3, 4). Till date, detailed mechanisms underlying the progression of AKI to CKD are poorly understood, and effective treatments for renal tubulointerstitial fibrosis following AKI are limited. Hence, novel approaches that attenuate renal tubulointerstitial fibrosis are urgently needed (5).

Hydroxychloroquine (HCQ), which was used initially as an antimalarial drug, was found to have beneficial effects on several immune diseases (6). The effects of HCQ, which include inhibition of lysosomal activity, inhibition of toll-like pattern recognition receptors (TLRs), and inhibition of RIG-I–stimulated induction of interferons, are multifaceted. Recently, HCQ was confirmed to be associated with a lower risk of incident CKD in users compared to non-users in patients with rheumatoid arthritis; however, little is known about the role of HCQ in renal tubulointerstitial fibrosis (7, 8). Although it seems that the use of HCQ is associated with benefits in some renal diseases, the mechanisms of HCQ need to be further explored (9–11).

TLRs, which serve as targets of HCQ, are pivotal components of the innate immune response and are expressed by macrophages and other immune cells (12). Although the mechanisms of action of HCQ in diseases are complex, the most important molecular targets of HCQ are TLRs. HCQ can inhibit the expression of TLR-7 and TLR-9, thereby contributing to the regulation of inflammatory responses. Recently, we found that depletion of TLR-9 could attenuate renal tubulointerstitial fibrosis; however, whether HCQ could serve as a TLR-9 inhibitor and attenuate CKD still remains to be elucidated (13).

To resolve the unsolved puzzle regarding the role of HCQ in renal tubulointerstitial fibrosis, in this study we utilized a traditional rodent model of renal tubulointerstitial fibrosis to further explore the biological effects of HCQ, especially on macrophages. A series of *in vivo* and *in vitro* assays were performed to explore the relationships among HCQ, macrophages, and renal fibrosis. A TLR-9 knockout (KO) mouse was constructed to further elucidate the underlying mechanisms of HCQ in CKD development following ischemia-reperfusion injury (IRI). Our results suggest that HCQ inhibits macrophage activation and MAPKs *via* the TLR-9 pathway, thereby attenuating renal fibrosis after IRI.

## Materials and Methods

### Animals and Ethics Statement

This study was performed in accordance with the animal welfare guidelines laid down in China (Laboratory Animal Guidelines for Ethical Review of Animal Welfare, GB/T 35892-2018) and approved by the Institutional Animal Care and Use Committee of Sun Yat-Sen University. Wildtype (WT) C57BL/6J mice were obtained from Charles River Laboratories (Beijing, China) and TLR-9 deficient (TLR-9^−/−^) mice with a C57BL/6J congenic background were provided by S.G. Wan (Gannan Medical University). Only male mice aged 6 to 8 weeks were used in subsequent experiments. Sources and confirmation of TLR-9 KO mice are provided in Supplementary Materials (Detailed methods and **Figure S1**).

### Induction of IRI in Mice

IRI was induced in male mice using an established method (13, 14). Mice were randomized placed either in an IRI or sham group (details of the number of mice per group can be found in the related paragraph). The bilateral IRI (bIRI) model was used for the IRI group. Mice were euthanized using isoflurane before surgery or on days 1, 7, 14, 21, or 28 post-surgery. Renal and blood samples were harvested for subsequent analyses. For HCQ (S4430, Selleck) treatment, HCQ was dissolved in carboxymethyl cellulose (CMC) and gavages were performed 30 min after induction of IRI for 7 days. The concentrations of HCQ used for treatment were 5, 10, and 20 mg/kg/day. The effects of 5 mg/kg/day HCQ were not significant, and no significant difference was found between 10 mg/kg/day HCQ and 20 mg/kg/day HCQ; hence, a final concentration of 10 mg/kg/day was used (data not shown).

### Measurement of Renal Function

Blood samples were first centrifuged at 2,000 ×*g* for 10 min at 4°C and then at 8,000 × *g* for 10 min at 4°C. Serum was collected and frozen at −80°C until use. Creatine (Cr) and blood urea nitrogen (BUN) levels were measured using an automatic biochemistry analyzer (7020; Hitachi, Tokyo, Japan).

### Renal Histology and Fibrosis Assessment

Renal tissues were retrieved without perfusion and fixed in 4% paraformaldehyde. Kidney paraffin sections (4 μm) were stained with Hematoxylin and Eosin (H&E) and Periodic Acid-Schiff (PAS) to assess renal injury. Sirius Red (SR) and Masson’s trichome (MT) staining were used for assessment of fibrosis.

### Immunohistochemistry and Immunofluorescence

Renal tissues were first perfused with cold saline (0.9%) followed by fixation with 4% paraformaldehyde. Paraffin-embedded slices (4 μm) were subjected to immunohistochemical staining as described in our previous study (15). Antibodies against fibronectin (FN; Ab2413; RRID: AB_2262874; Abcam), collagen I (COL I, Ab88147; RRID: AB_2081873; Abcam), α-smooth muscle actin (α-SMA; Ab32575; RRID: AB_722538; Abcam), and E-cadherin (E-Cad, Ab76319; RRID: AB_2076796; Abcam) were used to assess fibrosis and epithelial-to-mesenchymal transition (EMT). TLR-9 antibody (Ab53396; RRID: AB_883065; Abcam) was used to detect TLR-9, and cytokeratin 18 antibody (CK18; Ab668; RRID: AB_305647; Abcam) was used for verification of renal tubular epithelial cells (RTECs) (**Figure S2**).

### Real-Time Quantitative PCR (RT qPCR)

The RNAeasy™ Animal RNA Isolation Kit with Spin Column (R0024; Beyotime, Shanghai, China) was used to obtain total RNA from fresh tissues according to the manufacturer’s guidelines. Mean fold changes were calculated by averaging three duplicate measurements, and the gene expression results were normalized to *Gapdh*. Relative gene expressions in treated groups were compared with those in blank or sham groups. The 2^−△△CT^ method was used for calculation. The sequences of the primer pairs are listed in **Table 1**. Transcript-specific primers were generated from GenBank and primer specificity was verified using the NCBI Primer Blast. For Real-Time qPCR, 2 μg RNA was used for reverse transcription using PrimeScript ™ RT Master Mix (RR036A; Takara, Shiga, Japan); amplification was performed using SYBR Green Master Mix (Roche; Bruxelles, Belgium) and the LightCycler480 system (Roche).

**Table 1:** Primer sequences for Real-time quantitative PCR.

Transcript	Seq ID	Forward 5′ to 3′	Reverse 5′ to 3′
IL-1β	NM_008361	GAAATGCCACCTTTTGACAGTG	TGGATGCTCTCATCAGGACAG
IL-6	NM_031168	TAGTCCTTCCTACCCCAATTTCC	TTGGTCCTTAGCCACTCCTTC
TNF-α	NM_013693	CCCTCACACTCAGATCATCTTCT	GCTACGACGTGGGCTACAG
TGF-β1	NM_011577	CTCCCGTGGCTTCTAGTGC	GCCTTAGTTTGGACAGGATCTG
CD86	NM_019388	TGTTTCCGTGGAGACGCAAG	TTGAGCCTTTGTAAATGGGCA
CD206	NM_008625	CTCTGTTCAGCTATTGGACGC	CGGAATTTCTGGGATTCAGCTTC
IL-4	NM_021283	GGTCTCAACCCCCAGCTAGT	GCCGATGATCTCTCTCAAGTGAT
IL-10	NM_010548	GCTCTTACTGACTGGCATGAG	CGCAGCTCTAGGAGCATGTG
MIP-2	/	TTCCTGCTGTTTCTCTTACACCT	CTGTCTGCCTCTTTTGGTCAG
MCP-1	NM_011333	ACCTGCTGCTACTCATTCAC	TTGAGGTGGTTGTGGAAAAG
Arg-1	NM_007482	AGGAGCTGTCATTAGGGACATC	CTCCAAGCCAAAGTCCTTAGAG
Icam-1	NM_010493	TGTTTCCTGCCTCTGAAGC	CTTCGTTTGTGATCCTCCG
Vcam-1	NM_011693	AGTTGGGGATTCGGTTGTTCT	CCCCTCATTCCTTACCACCC
Smad 1	NM_008539	GCTTCGTGAAGGGTTGGGG	CGGATGAAATAGGATTGTGGGG
MyD88	NM_010851	TCATGTTCTCCATACCCTTGGT	AAACTGCGAGTGGGGTCAG
Snail 1	/	CACACGCTGCCTTGTGTCT	GGTCAGCAAAAGCACGGTT
TLR-9	NM_031178	CCAGTTTGTCAGAGGGAGCC	GGACAGGTGGACGAAGTCAG
Gapdh	NM_008085	AATGGATTTGGACGCATTGGT	TTTGCACTGGTACGTGTTGAT

### Flow Cytometry

Flow cytometry was performed as previously described (16). Anti-mouse CD16/CD32 (553141; RRID: AB_394656, clone 2.4G2; BD Biosciences, San Jose, CA, USA) was used for blocking nonspecific Fc sites. The antibodies used in this assay were acquired from BioLegend (San Diego, CA, USA). BV421 anti-mouse CD45 (RRID: AB_2562559), APC anti-mouse F4/80 (RRID: AB_893481), PE anti-mouse CD206 (MMR) (RRID: AB_10895754), FITC anti-mouse CD86 (RRID: AB_313149), and PerCP/Cyanine5.5 anti-mouse/human CD11b (RRID: AB_893232) antibodies were employed for detection of immune cells. Annexin V-FITC Apoptosis Detection Kit was used for apoptosis detection (Thermo Fisher Scientific Cat# BMS500FI/100, RRID: AB_2575598). Data were acquired on a FACS Calibur cytometer (Becton Dickinson [BD], Bedford, MA, USA) and analyzed using FlowJo software (Tree Star, Ashland, OR, USA).

### Isolation of Primary RTE Cells

RTE cells were extracted from adult male mice (6–8 weeks of age) following the method described by Ichimura et al. (17). Detailed methods can be found in the Supplementary Materials. For TGF-β culture, the second passage of RTE cells was used for subsequent assays. RTE cells isolated from WT and KO mice were exposed to TGF-β1 (10 ng/ml) (BioLegend, cat: 763102) for 72 h. Results were acquired from three independent assays.

### Isolation of Bone Marrow–Derived Macrophages

Bone marrow–derived macrophages (BMDMs) were generated using an established method (18). Cells generated from mouse femur were cultured with macrophage colony-stimulating factor (M-CSF; 25 ng/ml; Novoprotein, China) for 7 days. To achieve polarization of BMDMs, BMDMs were stimulated with IL-4 (20 ng/ml, Novoprotein, China), IL-13 (20 ng/ml, Novoprotein, China) or Lipopolysaccharides (LPS) (250 ng/ml, Sigma), and IFN-γ (20 ng/ml, Sigma) for 24 h.

### Cellular Immunofluorescence

For cellular immunofluorescence, cells were washed three times with phosphate-buffered saline (Sigma) to remove the culture medium. Fixation and permeability were performed for 15 min with 4% paraformaldehyde (P0099, Beyotime) for 20 min with 1% Triton X-100 (P0096, Beyotime). QuickBlock™ Blocking Buffer for Immunol Staining (P0260, Beyotime) was used to block non-specific antigens. Primary antibodies were used according to the objectives of each experiment, and the cells were incubated in appropriate antibodies overnight. Nuclear staining was performed using 4,6-diamidino-2-phenylindole (DAPI; P0131; Beyotime).

### Western Blotting Assay

After perfusion with cold saline (0.9%), proteins from renal tissue were acquired using a tissue homogenizer (KZ-II; Servicebio). A total of 20 μg of protein was used for WB analysis, and 10% SDS-PAGE gels were used for WB. Polyvinylidene fluoride membranes (0.45 µm; IPVH00010; Millipore, Billerica, MA) were used for protein transfer. Primary antibodies used for WB were purchased from Cell Signaling Technology (CST). Antibodies, such as SAPK/JNK (9252; RRID: AB_2250373; CST), Phospho-SAPK/JNK (Thr183/Tyr185) (81E11) (4668; RRID: AB_823588; CST), p38 MAPK (D13E1) (8690; RRID: AB_10999090; CST), Phospho-p38 MAPK (Thr180/Tyr182) (4511; RRID: AB_2139682; CST), p44/42 MAPK (Erk1/2) (4695; RRID: AB_390779; CST), and phospho-p44/42 MAPK (Erk1/2) (Thr202/Tyr204) (4370; RRID: AB_2315112; CST) were used to detect MAPK signaling pathways. GAPDH (D16H11) XP^®^ Rabbit mAb (HRP Conjugate) antibody was used for GAPDH detection (8884; RRID: AB_11129865; CST).

### Histology, Immunohistochemistry, and Immunofluorescence Analysis

Quantification of fibrosis, immunohistochemistry, and immunofluorescence analysis was performed using ImageJ software (NIH Image, Bethesda, MD, USA). Five randomly chosen high-resolution images per mouse (original magnification ×100) captured using the Leica system were used for quantification. Two independent investigators, blinded to the experimental conditions, performed this process.

### Statistics

The Kolmogorov-Smirnov (KS) normality test was first used to test whether the data were normally distributed; all the data met this criterion. Student’s t-test (two groups) and one-way analysis of variance (for ≥3 groups) were used to analyze significant differences. All values are expressed as the mean ± standard deviation (SD). Data were analyzed using GraphPad Prism 8.0 (GraphPad Software Inc., La Jolla, CA, USA). A P-value<0.05 was considered statistically significant.

## Results

### HCQ Reduces Renal Tubulointerstitial Fibrosis Following IRI *In Vivo*

**Figure 1:**
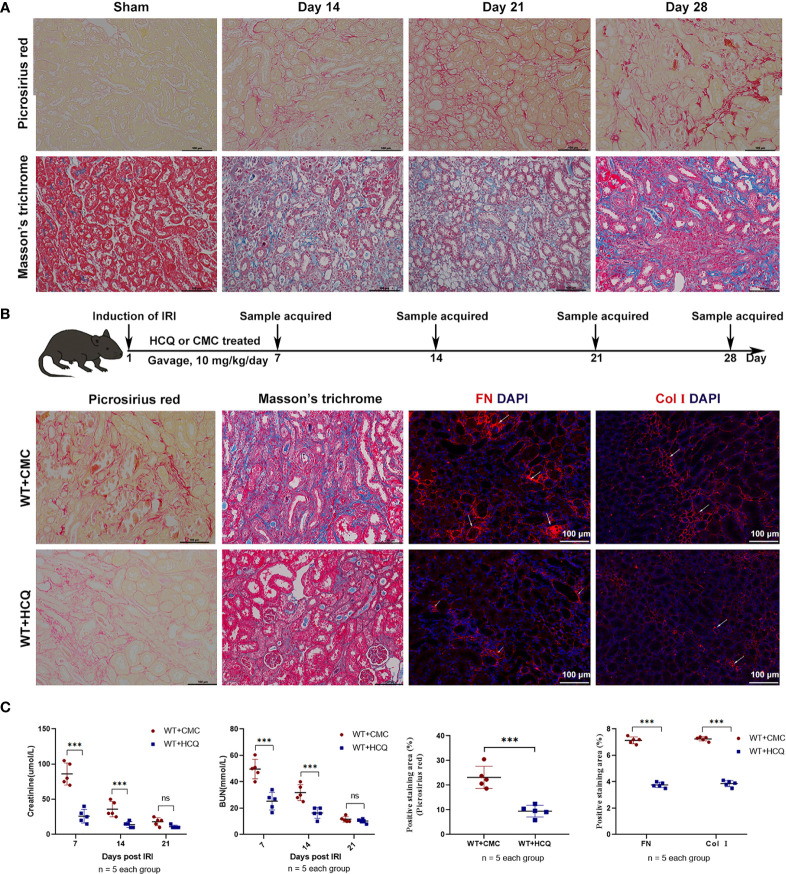
HCQ treatment reduces renal tubulointerstitial fibrosis following IRI *in vivo*. Mice were subjected to IRI and treated with either CMC or HCQ (10 mg/kg/day for 7 days). Serum samples and kidneys were harvested at various days following reperfusion. Tissue sections are representative of five mice per group. **(A)** Representative images of Sirius red and Masson’s trichrome stains on normal and injured kidneys of WT mice at various time points following reperfusion. The scale bars represent 100 μm. **(B)** Upper panel: Design of HCQ intervention and samples acquired; Down panel: Representative images of Picrosirius red - and Masson’s trichrome-treated FN and Col I at day 28 following reperfusion in CMC-treated and HCQ-treated groups for WT mice; the scale bars represent 100 μm. **(C)** Left panel: levels of serum creatinine and BUN from CMC-treated and HCQ-treated WT mice at various time-points following reperfusion; right panel: quantification of Sirius red-stained areas in sections of murine renal cortical medullary junction and semiquantitative analysis of COL I and FN immunofluorescence staining. Data are represented as means ± SD and analyzed using the Student’s t test. Magnification: 200×. NS, no significant difference, ***P < 0.001.

To construct a renal tubulointerstitial fibrosis model, WT mice were first used to examine the development of AKI in CKD (19). Renal tissues of WT mice subjected to bilateral clamping of renal arteries and veins for 26 min were obtained. Picrosirius red (PSR) and Masson’s trichrome (MT) were used to assess tubulointerstitial fibrosis on days 14, 21, and 28 post-injury. The degree of positive PSR- and MT-stained areas increased in a time-dependent manner, which indicated the successful construction of a renal tubulointerstitial fibrosis model after acute injury (**Figure 1A**). The effects of HCQ were further explored, and results suggested that renal fibrosis was attenuated in the HCQ-treated WT group than in CMC-treated WT group (**Figures 1A, B**) (P <0.01). Expression of FN and COL I was significantly inhibited in the HCQ-treated WT group compared to that in CMC-treated WT group (P <0.01) (**Figures 1B, C**). Renal function at days 7 and 14 was better preserved in HCQ-treated mice, whereas the difference was less apparent by day 21 post-IRI (**Figure 1C** and **Figure S3**).

### HCQ Treatment Inhibits Leukocyte and Macrophage Infiltration in the Kidney Following IRI *In Vivo*

**Figure 2:**
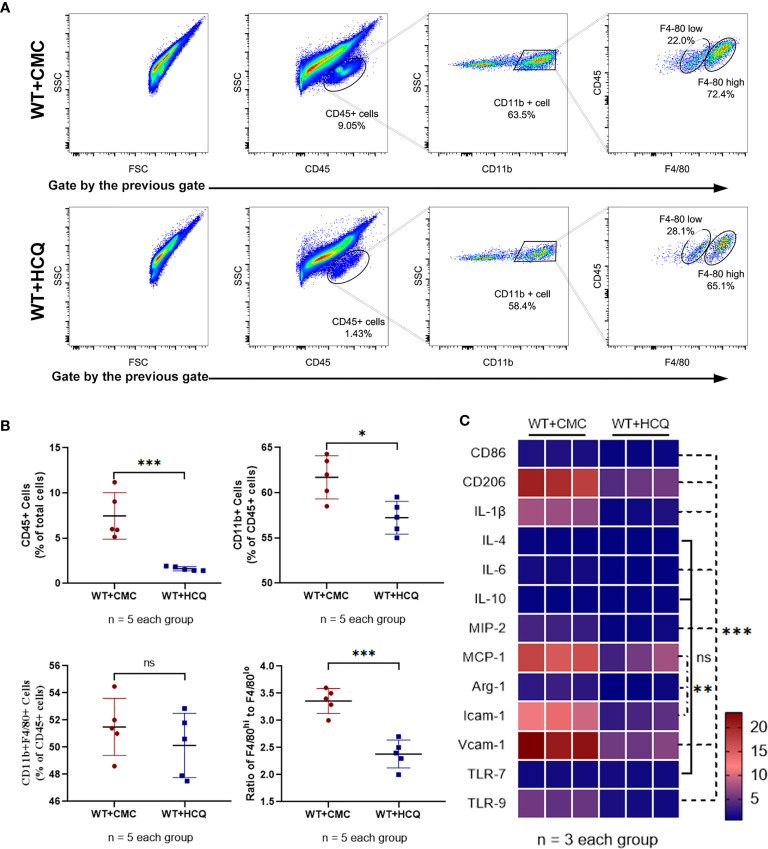
HCQ treatment regulates leukocyte infiltration and phenotype of macrophage in the kidney following IRI *in vivo*. Inflammatory cell infiltration in CMC-treated and HCQ-treated kidneys at day 28 following IRI was determined using flow cytometry. **(A)** Representative dot plots of CD45+ cells and different types of macrophages (CD11b^+^F4/80^high^ and CD11b^+^F4/80^low^). **(B)** Quantification of related immune cell infiltration, including CD 45+ cells, F4/80^high^, and F4/80^low^ subsets in CD11b+. **(C)** Heatmap of mRNA expressions related to macrophages in renal tissue at day 28 post IRI. Data are represented as means and analyzed using the Student’s t test. NS, no significant difference, *P < 0.05, **P < 0.01, ***P < 0.001.

Leukocyte infiltration is an important hallmark of inflammation during tissue injury (20). Infiltration of leukocytes, especially macrophages, which substantially contribute to IRI, was observed. Results showed that HCQ-treated mice exhibited significantly fewer CD45+ (total leukocyte) cells at day 28 post-IRI (7.46% vs. 1.63%, p = 0.001) (**Figure 2A**). Fewer macrophages were observed in the HCQ-treated WT group than in CMC-treated WT group post-injury (P<0.01) (**Figure 2A**). Macrophage phenotypes that contribute to the processes of fibrosis and tissue repair were also observed. CD11b^+^F4/80^high^ cells are regarded as M2-like macrophages, whereas CD11b^+^F4/80^low^ cells are M1-like macrophages (21, 22). The results showed that HCQ treatment decreased the number of M2-like macrophages to a greater extent than that of M1-like macrophages (**Figures 2A, B**). Cytokine and chemokine production-related M1 and M2 macrophages were also explored using RT qPCR, and the results were consistent with flow cytometry results. M1 macrophage-related (CD86, IL-1β, and IL-6) and M2 macrophages-related (CD206 and Arg-1) cytokines were inhibited in the HCQ-treated WT group, as also cytokines associated with migration (MIP-2, MCP-1, Icam-1, and Vcam-1) in renal tissue post-injury (**Figure 2C**). HCQ is a TLR inhibitor that can inhibit TLR-7 and TLR-9; therefore, the expression of TLRs was also assessed. Results showed that the expression of TLR-9 increased post-injury and was inhibited after HCQ treatment; however, no significant difference was found regarding the expression of TLR-7 (**Figure 2C**).

### HCQ Modulates Activation of Macrophages *In Vitro*

**Figure 3:**
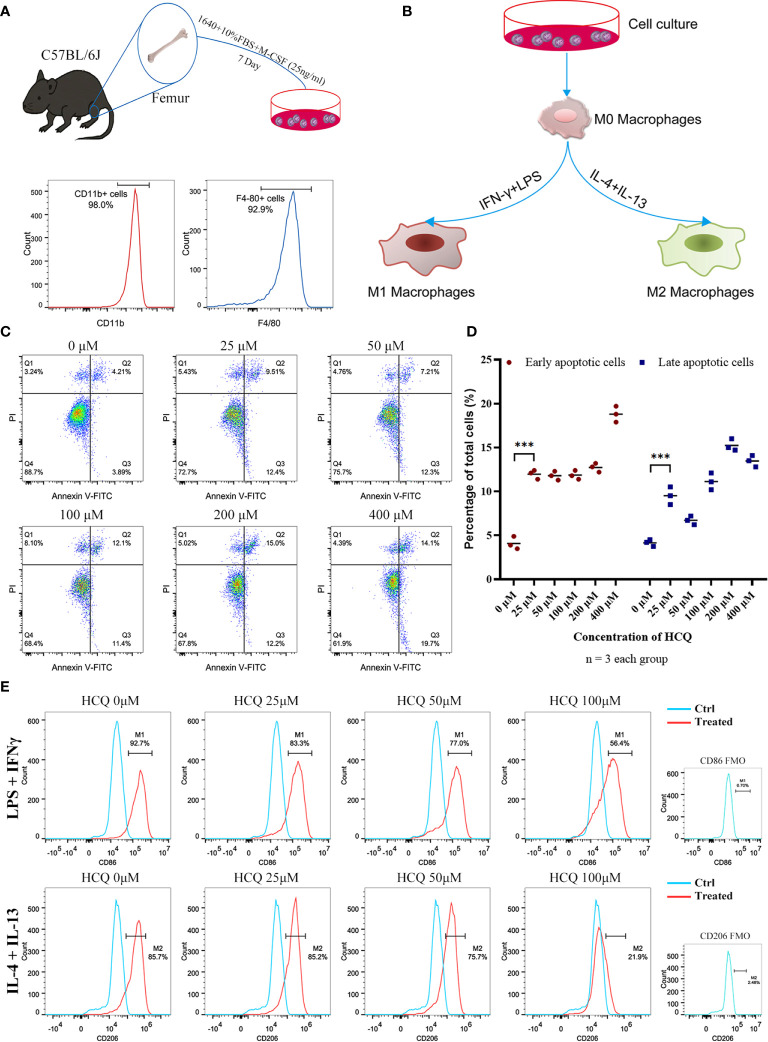
HCQ affects differentiation of BMDMs *in vitro*. **(A)** Models of BMDM acquisition and results of purity identification. **(B)** Models of polarization of BMDMs. **(C)** Apoptosis of BMDMs was assessed using Annexin V-FITC/PI Kit. Annexin V+ PI− represents early apoptotic cells, and Annexin V+ PI+ represents late apoptotic cells. **(D)** Quantitative analysis of related apoptotic cells. **(E)** Effects of HCQ on macrophage polarization. BMDMs were polarized into M1 or M2 subsets, and various concentrations of HCQ were added for 24 h to assess the effects of HCQ on the differentiation of macrophages. Data are represented as means and analyzed using the Student’s t test. ***P < 0.001.

To further explore the effects of HCQ on macrophages, BMDMs were acquired from the mouse femur and identified using CD11b and F4/80 (**Figure 3A**). M1 or M2 macrophages were induced using an established method (**Figure 3B**) (18, 23). Results suggested that the purity of macrophages (CD11b^+^F4/80^+^) was over 90% after co-culture with M-CSF (25 ng/ml) for 7 days. BMDMs were cultured with different doses of HCQ for 24 h, and the results indicated that HCQ promoted the apoptosis of BMDMs, including early apoptosis and late apoptosis (**Figures 3C, D**). Considering that the difference in apoptotic effects between 25 and 100 μM of HCQ was not significant, 25, 50, and 100 μM were used for further studies. Taken together, these results demonstrated that HCQ could inhibit the induction of M1 and M2 macrophages *in vitro* (**Figure 3E**).

### TLR-9 Knockout Limits the Inhibitory Effects of HCQ on BMDMs *In Vitro*

**Figure 4:**
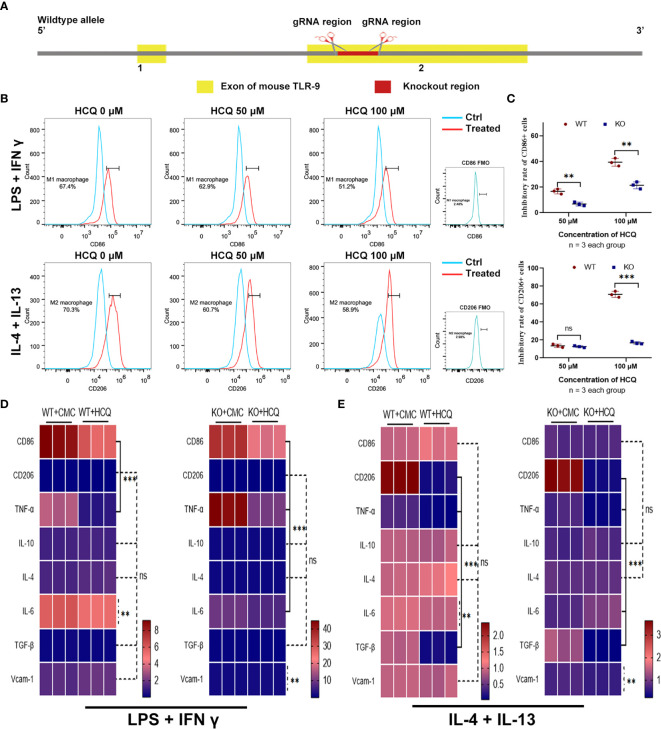
Effects of HCQ on differentiation of BMDMs are reduced after depletion of TLR-9 *in vitro*. **(A)** Overview of the target strategy. **(B)** Effects of HCQ on polarization of TLR-9^−/−^ macrophages. TLR-9^−/−^ BMDMs were polarized into M1 or M2 subsets, and various concentrations of HCQ were added for 24 h to assess the effects of HCQ on differentiation of macrophages. **(C)** Quantification of the rate of inhibition. **(D)** Heatmap of mRNA expressions related to macrophage polarization when stimulated with LPS + IFN-γ. **(E)** Heatmap of mRNA expressions related to macrophage polarization when stimulated with IL-4 + IL-13. Data are represented as means or means ± SD and analyzed using the Student’s t test. NS, no significant difference; **P < 0.01, ***P < 0.001.

HCQ is a TLR inhibitor that mainly inhibits the TLR-7 and TLR-9 pathways (12). Previous results indicated that the effects of HCQ on renal fibrosis may have relied on the inhibition of TLR-9 (**Figure 2C**); therefore, a TLR-9 KO mouse was constructed (**Figure 4A**). TLR-9 KO BMDMs were obtained from KO mice, and macrophage differentiation was induced using the same methods mentioned above. Upon induction, the proportions of M1 and M2 macrophages were found to be lower in KO BMDMs than in WT BMDMs (**Figure 4B**). The inhibitory effects of HCQ on proportions of CD86+ cells (M1) and CD206 cells (M2) were also observed. Proportions of M1- and M2-like macrophages were significantly reduced in KO group than in WT group (P<0.01), and it seemed that the proportion of M2 macrophages was reduced to a greater extent when treated with HCQ at the concentration of 100 μM (**Figure 4C**). Assessment of cytokine and chemokine production also indicated that HCQ could decrease the expression of CD86, TNF-α, IL-6, and Vcam-1 when stimulated with LPS and IFN-γ (**Figure 4D**). The expression of CD206, TNF-α, IL-6, TGF-β1, and Vcam-1 was also inhibited by HCQ when stimulated with IL-4 and IL-13 (**Figure 4E**).

### HCQ Attenuates Renal Tubulointerstitial Fibrosis Through TLR-9 Receptor *In Vivo*

**Figure 5:**
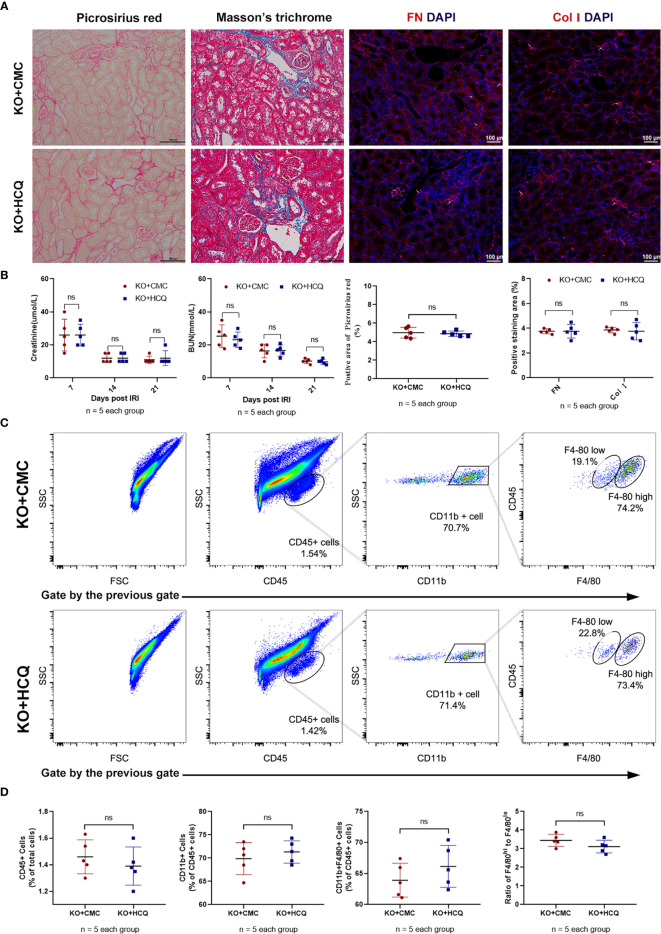
Effects of HCQ on renal tubulointerstitial fibrosis following IRI mainly rely on the TLR-9 signal pathway *in vivo*. KO mice were subjected to IRI and treated with either CMC or HCQ (10 mg/kg/day for 7 days). Serum samples and kidneys were collected 28 days post- reperfusion. Tissue sections are representative of five mice per group. **(A)** Representative images of Sirius red- Masson’s trichrome-staining of FN and Col I at day 28 following reperfusion in CMC-treated KO mice and HCQ treated-KO mice; the scale bars represent 100 μm. **(B)** Left panel: serum creatinine and urea from CMC-treated and HCQ-treated WT mice at various time- points following reperfusion; right panel: quantification of Sirius red-stained areas in murine renal cortical medullary junction sections and semiquantitative analysis of COL I and FN immunofluorescence staining; **(C)** Representative dot plots of CD45+ cells and different types of macrophage (CD11b^+^F4/80^high^ and CD11b^+^F4/80^low^). **(D)** Quantification of related immune cell infiltration, including CD 45+ cells, F4/80^high^, and F4/80^low^ subsets in CD11b+. Data are represented as means ± SD and analyzed using the Student’s t test. Magnification: 200×. NS, no significant difference.

KO mice were also used to further explore the effects of HCQ on CKD *in vivo*, and renal tissues were acquired 28 days post-IRI. Results showed that the improved effects of HCQ on the transition of AKI to CKD were eliminated in KO mice, and no significant difference in renal fibrosis or infiltration of leukocytes was found between HCQ- and CMC- treated KO groups (**Figure 5**).

### HCQ Down-Regulates Mitogen-Activated Protein Kinase Inflammatory Signaling Pathways During Renal Tubulointerstitial Fibrosis Vial TLR-9 *In Vivo*

**Figure 6:**
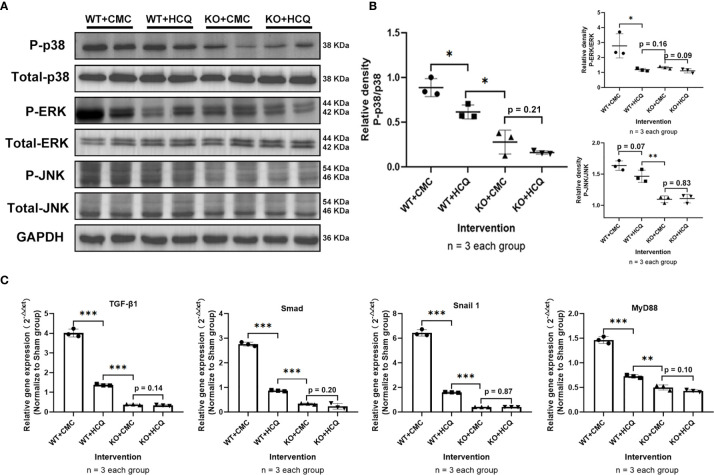
Depletion of TLR-9 attenuates renal fibrosis following IRI through the inhibition of MAPKs and pro-inflammatory chemokine/cytokine induction *in vivo*. Renal tissues were acquired on day 21 following IRI. **(A)** Activations of MAPKs. **(B)** Semiquantitative analysis of the activation of MAPKs. **(C)** Expression of pro-inflammatory chemokines/cytokines. Data are shown as means ± SD and analyzed using one-way analysis of variance. Each dot represents an individual mouse. *P < 0.05, **P < 0.01, ***P < 0.001. IRI, ischemia-reperfusion injury; WT, wild-type; KO, knockout; MAPK, mitogen-activated protein kinase; HCQ, hydroxychloroquine; CMC, carboxymethyl cellulose.

To further determine how HCQ might affect the inflammatory signaling pathways during renal fibrosis following IRI, kidneys were acquired at day 21 post-injury to evaluate the mitogen-activated protein kinase (MAPK) signaling pathway, a major contributor to fibrosis (24, 25). Phosphorylation of p38, Erk, and JNK decreased following HCQ treatment in the WT group, while no significant difference was found after HCQ treatment in the KO group (**Figures 6A, B**). Cytokines related to pro-fibrotic and TLR-9 signaling pathways were also explored. Levels of TGF-β1, Smad, Snail 1, and MyD88 were downregulated in the WT group when treated with HCQ, and no significant difference was found between HCQ- and CMC-treated KO groups (**Figure 6C**).

### TLR-9 Signal Pathways Do Not Contribute to Improvement of Partial Epithelial-to-Mesenchymal Transition (EMT) *In Vitro*

**Figure 7:**
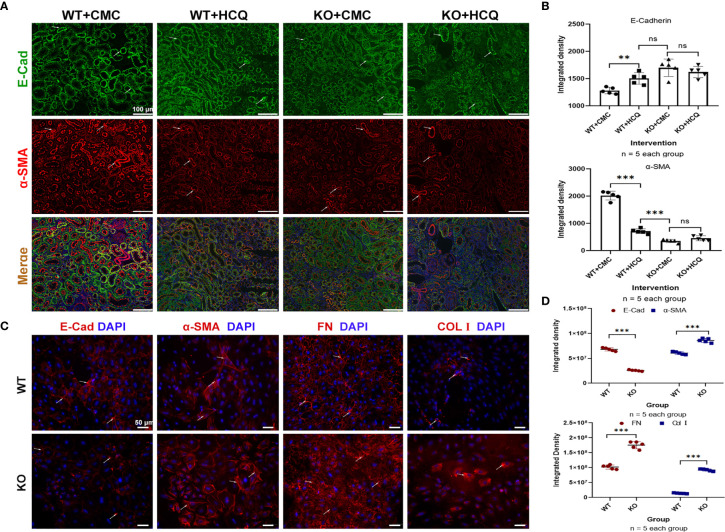
TLR-9 signal pathway did not contribute to the improvement of EMT *in vitro*. **(A)** First row: representative fluorescence microscope images of E-Cad (Green), α-SMA (Red), and DAPI (Blue) staining of the kidneys of WT and KO mice that were treated with CMC or HCQ following reperfusion; the scale bars represent 100 μm.; **(B)** Semiquantitative analysis of E-Cad and α-SMA immunofluorescence staining. **(C)** Immunofluorescence of COL I, FN, E-Cad, and α-SMA was used to assess fibrosis and EMT of RTECs after being exposed to TGF-β (10 ng/ml) for 72 h; the scale bars represent 100 μm. **(D)** Semiquantitative analysis of E-Cad, α-SMA, FN, and COL I immunofluorescence staining of RTECs following TGF-β treatment. Data are shown as means ± SD and analyzed using one-way analysis of variance or Student’s t test. Each dot represents an individual assay. Magnification: 200×. NS, no significant difference; **P < 0.01, ***P < 0.001. EMT, epithelial-to-mesenchymal transition; RTECs, renal tubular epithelial cells; WT, wild-type; KO, knockout; E-Cad, E-cadherin; α-SMA, α-smooth muscle; COL I, collagen I; FN, fibronectin.

Partial EMT, which leads to dedifferentiation of renal epithelial cells and promotes fibrosis, also plays a role in renal fibrosis (26, 27). Renal tissues were acquired at day 21 post-IRI, and the roles of TLR-9 signaling pathway in EMT were also explored. Expression of E-Cad (an epithelial marker) was significantly higher in the HCQ-treated WT group than in CMC-treated WT group *in vivo* (P < 0.01), whereas the expression of α-SMA (indicating a subset of activated fibrogenic cells) was lower in the HCQ-treated WT group than in CMC-treated WT group *in vivo* (P < 0.01) (**Figures 7A, B**). To further determine the effects of TLR-9 on RTECs, RTECs were isolated. A traditional EMT model involving treatment with TGF-β (10 ng/ml) was used (28). In contrast to the *in vivo* results, depletion of TLR-9 aggravated fibrosis (defined as high levels of FN and Col I, P < 0.001) and EMT (defined as low levels of E-Cad and high levels of α-SMA, P < 0.001) of RTECs *in vitro* (**Figures 7C, D**).

## Discussion

**Figure 8:**
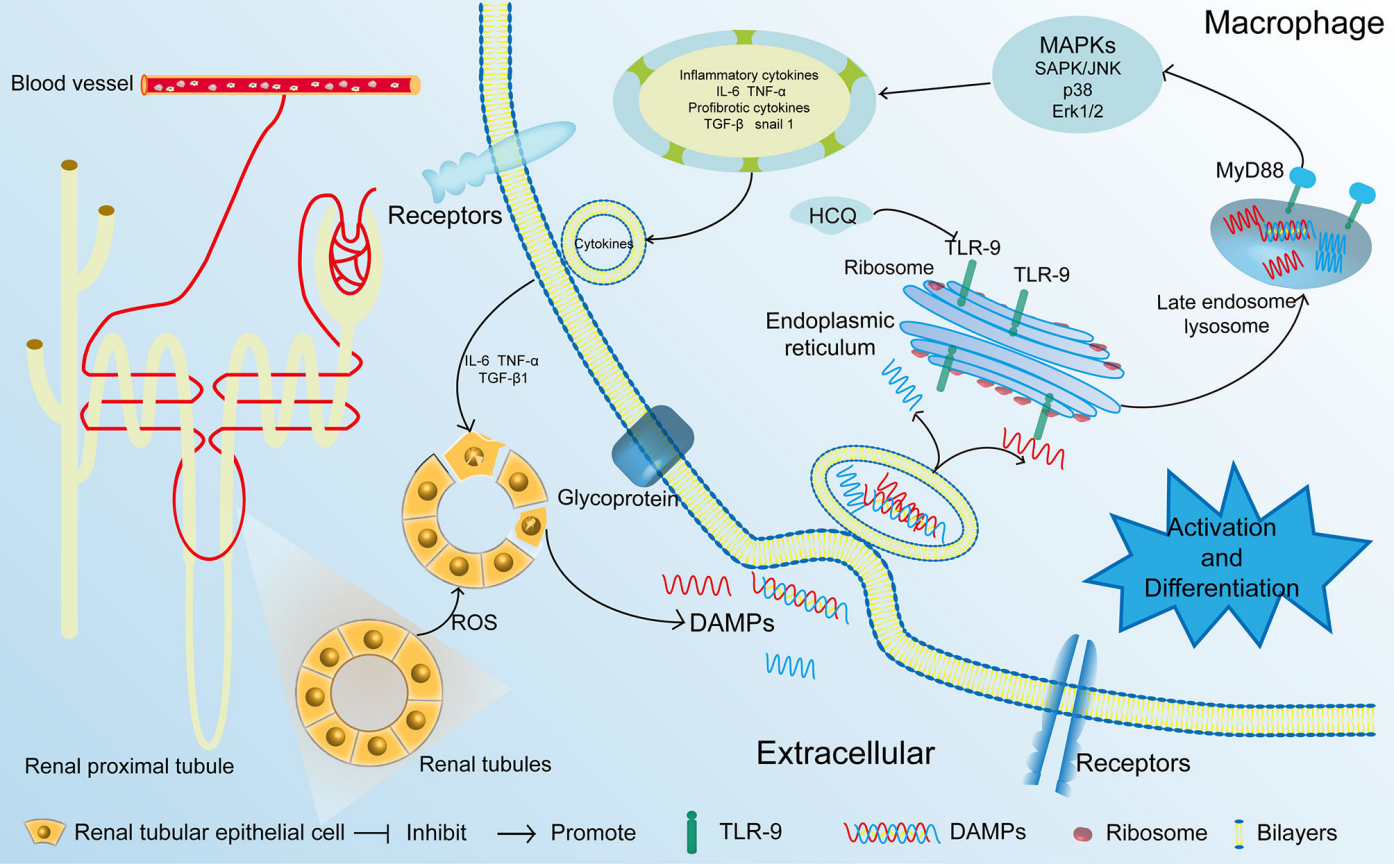
Proposed mechanisms of HCQ function in renal fibrosis following IRI. After being exposed to ischemia and reperfusion, RTECs were damaged. DAMPs that were released from the damaged RTECs flowed into blood vessels and activated monocytes. DAMPs also activated the TLR-9/MyD88 signal pathway, thereby activating MAPKs. Inflammatory and profibrotic cytokines including IL-6, TNF α, TGF-β were synthesized and released, resulting in the aggravation damage and fibrosis of kidney. RTECs, renal tubular epithelial cells; TLR-9, toll-like receptor-9; IRI, ischemia-reperfusion injury; DAMP, damage-associated molecular pattern.

IRI is a major cause of AKI that leads to CKD (5, 29). In the present study, we first identified that HCQ served as a TLR-9 inhibitor and inhibited the activation of macrophages, thereby attenuating renal fibrosis post-IRI. Treatment with HCQ decreased intrarenal infiltration of macrophages, especially M-2 macrophages, and reduced the severity of inflammation during renal tubulointerstitial fibrosis *in vivo*. However, the effects of HCQ on renal fibrosis or activation of macrophages decreased after TLR-9 depletion (**Figure 8**).

HCQ was first approved in the United States for the therapy and prevention of malaria in 1955. The effects of HCQ are complex, and it can not only serve as an immunomodulatory molecule, but also as an autophagy modulator (30). The functions of HCQ have been explored in some renal diseases, including IRI and IgAN (9, 11), but little is known about the effects of HCQ on renal tubulointerstitial fibrosis. In this study, we focused on the effects of HCQ on TLRs. Expression of TLR-9 but not TLR-7 in renal tissue post-IRI was inhibited by HCQ, and the results are consistent with our previous study showing that depletion of TLR-9 attenuated renal fibrosis. Previous results indicated that the use of HCQ was associated with a lower rate of incidence of CKD in rheumatoid arthritis patients (8). Our study demonstrated direct effects and mechanisms of HCQ on renal tubulointerstitial fibrosis.

TLRs are major sensors of the innate immune system and mediate inflammation and tissue regeneration (31). TLR-9, a member of the toll-like receptor family, also serves as a target of HCQ (32). Because HCQ attenuates renal fibrosis and inhibits activation of macrophages, we hypothesized that the effects of HCQ on renal tubulointerstitial fibrosis mainly rely on TLR-9. A TLR-9 KO mouse was constructed to further confirm our hypothesis. *In vivo* and *in vitro* results suggest that the effects of HCQ on macrophages and renal fibrosis decreased after depletion of TLR-9, and HCQ could not attenuate renal fibrosis post-IRI in the KO group. These results highlight that HCQ attenuates kidney fibrosis through the TLR-9 signaling pathway. Taking into account that it is difficult to distinguish between the effects of TLR-9 on CKD from those on AKI, effects of TLR-9 on AKI were also explored. The results revealed that TLR-9 was not required for the recovery of early renal function following IRI. Moreover, no significant difference was found in the levels of serum Cr or BUN between TLR-9 KO and WT mice or renal tubular injury scores at 24 and 48 h (Data not shown). These results also indicate TLR-9 could be an ideal therapeutic target for CKD.

TLR-9 is widely expressed in multiple cells, including macrophages, dendritic cells, B lymphocytes, and some interstitial tissues (33). It is primarily located in endosomes and recognizes viruses, endogenous nucleic acids, and bacteria. Following IRI injury, damage-associated molecular patterns are released and sensed by these TLR-9—expressed cells, especially macrophages (34, 35). In this study, we focused on the effects of HCQ on macrophages during CKD to further determine the relationship between HCQ and macrophages and tubulointerstitial fibrosis. *In vivo* and *in vitro* results suggest that HCQ inhibits the activation and differentiation of macrophages. A low concentration (50 μM) of HCQ decreased the differentiation of macrophages into an M1-like phenotype, while a high concentration (100 μM) of HCQ reduced differentiation of M2-like macrophages to a greater extent. M1-like macrophages mainly participate in acute inflammatory response and mediate ROS-induced tissue damage, including IRI. These results are consistent with previous results, which indicate that HCQ can attenuate renal IRI injury (11). M2-like macrophages are recognized to be anti-inflammatory and may also lead to fibrosis post-injury. Hence, inhibition of M1-like and M2-like macrophages by HCQ not only attenuates the inflammatory response during AKI, but also inhibits renal fibrosis.

Proinflammatory and profibrotic cytokines can also accelerate the progression of fibrosis (36). Activation of TLR-9 can induce several signaling pathways, especially those involving MAPKs. Moreover, proinflammatory and profibrotic cytokines, including IL-1β, IL-6, TNF-α, and TGF-β, can also be released (37, 38). Previous studies have shown that activation of MAPKs could directly contribute to fibroblast activation and renal fibrosis (39); these results were also confirmed in the present study. Consistent with the histological and immunohistochemical results, we found that inflammation and fibrosis signaling pathways were inhibited by HCQ and terminated after depletion of TLR-9.

Recently, increasing attention has been paid to the effects of renal intrinsic cells, especially RTECs, on fibrosis and inflammation. RTECs have been shown not only to contribute to the aggravation of inflammation, but also to take part in EMT (26, 27). Notably, TLR-9 deficiency did not lead to better preservation of RTECs; rather, fibrosis and EMT in TLR-9 KO RTE cells were indicative of more severe injury. These results also provide evidence that the effects of HCQ on renal fibrosis do not rely on the improvement of EMT. Inhibition of infiltrating lymphocytes and reduced pro-inflammatory cytokines may account for the improvement in EMT *in vivo*.

This study, nevertheless, has a few limitations. First, although the effects of HCQ on macrophages have been investigated *in vitro*, we focused only on the effects of HCQ on macrophages and inflammation. Other effects of HCQ on fibrosis should also be explored. Second, M2 macrophages can be divided into various subsets, including M2a, M2b, M2c, and M2d; detailed effects of HCQ on such macrophage subsets should be further explored. Finally, because the effects of HCQ were observed only in animal studies and HCQ has been used clinically, clinical trials should be performed to further confirm the effect of HCQ on patients with CKD.

Overall, the results of this study provide novel insights into the roles of HCQ in acute and chronic kidney injury. HCQ treatment attenuated renal fibrosis following renal injury *in vivo*, whereas the effects of HCQ could be terminated by depletion of TLR-9. Our results also provide new evidence that HCQ could be an ideal TLR-9 inhibitor for the treatment of renal tubulointerstitial fibrosis.

## Data Availability Statement

The original contributions presented in the study are included in the article/**Supplementary Material**. Further inquiries can be directed to the corresponding author.

## Ethics Statement

The animal study was reviewed and approved by the Institutional Animal Care and Use Committee of Sun Yat-Sen University. Written informed consent was obtained from the owners for the participation of their animals in this study.

## Author Contributions

HZ and QS conceived and designed the experiments. HZ, YZ, and JH performed experiments with the help of ZY, RZ, LL, ZL, and YY, HZ, and YZ analyzed the data and drafted the manuscript. All authors contributed to the article and approved the submitted version.

## Funding

This study was supported by the National Natural Science Foundation of China (grants 81970650, 81770753, 81800662), National Key R&D Program of China (grant numbers 2018YFA0108804), and The PhD Start-up Fund of Natural Science Foundation of Guangdong Province, China (grant 2018A030310324).

## Conflict of Interest

The authors declare that the research was conducted in the absence of any commercial or financial relationships that could be construed as a potential conflict of interest.

## References

[1] Li L Kang H Zhang Q D’Agati VD Al-Awqati Q Lin F. FoxO3 activation in hypoxic tubules prevents chronic kidney disease. J Clin Invest (2019) 129(6):2374–89. 10.1172/JCI122256 PMC6541430 30912765

[2] Kishi S Brooks CR Taguchi K Ichimura T Mori Y Akinfolarin A. Proximal tubule ATR regulates DNA repair to prevent maladaptive renal injury responses. J Clin Invest (2019) 129(11):4797–816. 10.1172/JCI122313 PMC6819104 31589169

[3] Bonventre JV Yang L. Cellular pathophysiology of ischemic acute kidney injury. J Clin Invest (2011) 121(11):4210–21. 10.1172/JCI45161 PMC3204829 22045571

[4] Palomba H Castro I Yu L Burdmann EA. The duration of acute kidney injury after cardiac surgery increases the risk of long-term chronic kidney disease. J Nephrol (2017) 30(4):567–72. 10.1007/s40620-016-0351-0 27704389

[5] Forni LG Darmon M Ostermann M Oudemans-van Straaten HM Pettila V Prowle JR. Renal recovery after acute kidney injury. Intensive Care Med (2017) 43(6):855–66. 10.1007/s00134-017-4809-x PMC5487594 28466146

[6] Muller-Calleja N Manukyan D Canisius A Strand D Lackner KJ. Hydroxychloroquine inhibits proinflammatory signalling pathways by targeting endosomal NADPH oxidase. Ann Rheum Dis (2017) 76(5):891–7. 10.1136/annrheumdis-2016-210012 27903507

[7] Bethel M Yang FM Li S Nahman NS Oliver AM Machua W. Hydroxychloroquine in patients with systemic lupus erythematosus with end-stage renal disease. J Investig Med (2016) 64(4):908–10. 10.1136/jim-2016-000065 26911274

[8] Wu CL Chang CC Kor CT Yang TH Chiu PF Tarng DC. Hydroxychloroquine Use and Risk of CKD in Patients with Rheumatoid Arthritis. Clin J Am Soc Nephrol (2018) 13(5):702–9. 10.2215/CJN.11781017 PMC5969483 29661770

[9] Liu LJ Yang YZ Shi SF Bao YF Yang C Zhu SN. Effects of Hydroxychloroquine on Proteinuria in IgA Nephropathy: A Randomized Controlled Trial. Am J Kidney Dis (2019) 74(1):15–22. 10.1053/j.ajkd.2019.01.026 30922594

[10] Bai L Li J Li H Song J Zhou Y Lu R. Renoprotective effects of artemisinin and hydroxychloroquine combination therapy on IgA nephropathy via suppressing NF-kappaB signaling and NLRP3 inflammasome activation by exosomes in rats. Biochem Pharmacol (2019) 169:113619. 10.1016/j.bcp.2019.08.021 31465776

[11] Tang TT Lv LL Pan MM Wen Y Wang B Li ZL. Hydroxychloroquine attenuates renal ischemia/reperfusion injury by inhibiting cathepsin mediated NLRP3 inflammasome activation. Cell Death Dis (2018) 9(3):351. 10.1038/s41419-018-0378-3 29500339 PMC5834539

[12] Clancy RM Markham AJ Buyon JP. Endosomal Toll-like receptors in clinically overt and silent autoimmunity. Immunol Rev (2016) 269(1):76–84. 10.1111/imr.12383 26683146 PMC4685960

[13] Zheng H Zhang Y Li L Zhang R Luo Z Yang Z. Depletion of Toll-Like Receptor-9 Attenuates Renal Tubulointerstitial Fibrosis After Ischemia-Reperfusion Injury. Front Cell Dev Biol (2021) 9:641527. 10.3389/fcell.2021.641527 33644078 PMC7907438

[14] Kenwood BM Weaver JL Bajwa A Poon IK Byrne FL Murrow BA. Identification of a novel mitochondrial uncoupler that does not depolarize the plasma membrane. Mol Metab (2014) 3(2):114–23. 10.1016/j.molmet.2013.11.005 PMC3953706 24634817

[15] Liao T Zhang Y Ren J Zheng H Zhang H Li X. Noninvasive quantification of intrarenal allograft C4d deposition with targeted ultrasound imaging. Am J Transplant (2019) 19(1):259–68. 10.1111/ajt.15105 30171802

[16] Zhao D Li S Liao T Wei Y Liu M Han F. Triptolide inhibits donor-specific antibody production and attenuates mixed antibody-mediated renal allograft injury. Am J Transplant (2018) 18(5):1083–95. 10.1111/ajt.14602 29178433

[17] Ichimura T Asseldonk EJ Humphreys BD Gunaratnam L Duffield JS Bonventre JV. Kidney injury molecule-1 is a phosphatidylserine receptor that confers a phagocytic phenotype on epithelial cells. J Clin Invest (2008) 118(5):1657–68. 10.1172/JCI34487 PMC2293335 18414680

[18] Ma PF Gao CC Yi J Zhao JL Liang SQ Zhao Y. Cytotherapy with M1-polarized macrophages ameliorates liver fibrosis by modulating immune microenvironment in mice. J Hepatol (2017) 67(4):770–9. 10.1016/j.jhep.2017.05.022 28596109

[19] Fu Y Tang C Cai J Chen G Zhang D Dong Z. Rodent models of AKI-CKD transition. Am J Physiol Renal Physiol (2018) 315(4):F1098–F106. 10.1152/ajprenal.00199.2018 PMC6230729 29949392

[20] Ernandez T Mayadas TN. The Changing Landscape of Renal Inflammation. Trends Mol Med (2016) 22(2):151–63. 10.1016/j.molmed.2015.12.002 PMC4738004 26778189

[21] Satoh T Takeuchi O Vandenbon A Yasuda K Tanaka Y Kumagai Y. The Jmjd3-Irf4 axis regulates M2 macrophage polarization and host responses against helminth infection. Nat Immunol (2010) 11(10):936–44. 10.1038/ni.1920 20729857

[22] Peng Q Wu W Wu KY Cao B Qiang C Li K. The C5a/C5aR1 axis promotes progression of renal tubulointerstitial fibrosis in a mouse model of renal ischemia/reperfusion injury. Kidney Int (2019) 96(1):117–28. 10.1016/j.kint.2019.01.039 31029505

[23] Mao L Kitani A Hiejima E Montgomery-Recht K Zhou W Fuss I. Bruton tyrosine kinase deficiency augments NLRP3 inflammasome activation and causes IL-1beta-mediated colitis. J Clin Invest (2020) 130(4):1793–807. 10.1172/JCI128322 PMC7108929 31895698

[24] Budas GR Boehm M Kojonazarov B Viswanathan G Tian X Veeroju S. ASK1 Inhibition Halts Disease Progression in Preclinical Models of Pulmonary Arterial Hypertension. Am J Respir Crit Care Med (2018) 197(3):373–85. 10.1164/rccm.201703-0502OC 28910144

[25] Fan J Zou L Cui K Woo K Du H Chen S. Atrial overexpression of angiotensin-converting enzyme 2 improves the canine rapid atrial pacing-induced structural and electrical remodeling. Fan, ACE2 improves atrial substrate remodeling. Basic Res Cardiol (2015) 110(4):45. 10.1007/s00395-015-0499-0 26143546 PMC7101981

[26] Lovisa S LeBleu VS Tampe B Sugimoto H Vadnagara K Carstens JL. Epithelial-to-mesenchymal transition induces cell cycle arrest and parenchymal damage in renal fibrosis. Nat Med (2015) 21(9):998–1009. 10.1038/nm.3902 26236991 PMC4587560

[27] Grande MT Sanchez-Laorden B Lopez-Blau C De Frutos CA Boutet A Arevalo M. Snail1-induced partial epithelial-to-mesenchymal transition drives renal fibrosis in mice and can be targeted to reverse established disease. Nat Med (2015) 21(9):989–97. 10.1038/nm.3901 26236989

[28] Su J Morgani SM David CJ Wang Q Er EE Huang YH. TGF-beta orchestrates fibrogenic and developmental EMTs via the RAS effector RREB1. Nature (2020) 577(7791):566–71. 10.1038/s41586-019-1897-5 PMC7450666 31915377

[29] Jang HR Rabb H. Immune cells in experimental acute kidney injury. Nat Rev Nephrol (2015) 11(2):88–101. 10.1038/nrneph.2014.180 25331787

[30] Lei ZN Wu ZX Dong S Yang DH Zhang L Ke Z. Chloroquine and hydroxychloroquine in the treatment of malaria and repurposing in treating COVID-19. Pharmacol Ther (2020) 216:107672. 10.1016/j.pharmthera.2020.107672 32910933 PMC7476892

[31] Fitzgerald KA Kagan JC. Toll-like Receptors and the Control of Immunity. Cell (2020) 180(6):1044–66. 10.1016/j.cell.2020.02.041 PMC9358771 32164908

[32] Gies V Bekaddour N Dieudonne Y Guffroy A Frenger Q Gros F. Beyond Anti-viral Effects of Chloroquine/Hydroxychloroquine. Front Immunol (2020) 11:1409. 10.3389/fimmu.2020.01409 32714335 PMC7343769

[33] Leemans JC Kors L Anders HJ Florquin S. Pattern recognition receptors and the inflammasome in kidney disease. Nat Rev Nephrol (2014) 10(7):398–414. 10.1038/nrneph.2014.91 24890433

[34] Huen SC Cantley LG. Macrophages in Renal Injury and Repair. Annu Rev Physiol (2017) 79:449–69. 10.1146/annurev-physiol-022516-034219 28192060

[35] Ren Q Cheng L Yi J Ma L Pan J Gou SJ. Toll-like Receptors as Potential Therapeutic Targets in Kidney Diseases. Curr Med Chem (2020) 27(34):5829–54. 10.2174/0929867325666190603110907 31161985

[36] Humphreys BD. Mechanisms of Renal Fibrosis. Annu Rev Physiol (2018) 80:309–26. 10.1146/annurev-physiol-022516-034227 29068765

[37] O’Keeffe M Grumont RJ Hochrein H Fuchsberger M Gugasyan R Vremec D. Distinct roles for the NF-kappaB1 and c-Rel transcription factors in the differentiation and survival of plasmacytoid and conventional dendritic cells activated by TLR-9 signals. Blood (2005) 106(10):3457–64. 10.1182/blood-2004-12-4965 16037393

[38] Kuo CH Hsieh CC Kuo HF Huang MY Yang SN Chen LC. Phthalates suppress type I interferon in human plasmacytoid dendritic cells via epigenetic regulation. Allergy (2013) 68(7):870–9. 10.1111/all.12162 23738920

[39] Inoue T Takenaka T Hayashi M Monkawa T Yoshino J Shimoda K. Fibroblast expression of an IkappaB dominant-negative transgene attenuates renal fibrosis. J Am Soc Nephrol (2010) 21(12):2047–52. 10.1681/ASN.2010010003 PMC3014017 20847140

