# Supplementary material for: Hydroxychloroquine Inhibits Macrophage Activation and Attenuates Renal Fibrosis After Ischemia-Reperfusion Injury
Source: Front Immunol. 2021 Apr 14;12:645100. doi: 10.3389/fimmu.2021.645100 (PMC8079743; doi:10.3389/fimmu.2021.645100)
Supplement: Supplementary file 1 [file DataSheet_1.docx]

**Supplementary MATERIAL**

## Hydroxychloroquine inhibits macrophage activation and attenuates renal fibrosis after ischemia-reperfusion injury

Haofeng Zheng†, Yannan Zhang†, Jiannan He†, Zhe Yang, Rui Zhang, Lei Li, Zihuan Luo, Yongrong Ye, and Qiquan Sun*

^†^H.Z., Y.Z. and J.H. contributed equally to this work.

Organ Transplantation Research Institute of Sun Yat-sen University, the Third Affiliated Hospital of Sun Yat-sen University, Guangzhou, China

**Corresponding author**

Qiquan Sun, Organ Transplantation Research Institute of Sun Yat-sen University, the Third Affiliated Hospital of Sun Yat-sen University, 2693 Kaichuang Avenue, Huangpu District, Guangzhou, Guangdong, China, 510630. Email: [sunqiq@mail.sysu.edu.cn](mailto:sunqiq@mail.sysu.edu.cn)

This article contains the following supplemental material online at:

Detailed methods.

Figure S1. Construction and confirmation of TLR-9 knockout mice.

Figure S2. Verification of RTECs in vitro.

Figure S3. HCQ treated accelerates chronic kidney disease recovery following IRI in vivo.

**Detailed methods**

**Animals**

C57BL/6J-TLR9 ^-/-^ mice were backcrossed at least 10 times onto the C57BL/6J strain. Mice were genotyped using PCR-based methods on DNA isolated from blood with the following primer pairs: TLR-9, Forward 1: 5′-ctg act tcg tcc acc tgtc-3′, Reverse 1: 5′- tct tca ggg gtg gct tct g-3′ and Reverse 2: 5′-ttc ttg tag tag cag ttc ccg-3′. All mice were housed with 12-hour light/dark cycles, with normal food provided *ad libitum*.

**Induction of IRI in Mice**

WT and KO mice were anesthetized using isoflurane (5% induction and 1% maintenance) and placed on a warm pad to maintain body temperature at 36 °C. Following midline abdominal incision, both renal pedicles were clamped using non-traumatic clamps (FT722T, Aesculap, Tuttlingen, Germany) for 26 minutes. Sham group mice were only subjected to abdominal incision.

**Isolation of Primary RTE Cells**

Collagenase solution (1 mg/ml; Sigma-Aldrich, St. Louis, MO, USA) was used for renal digestion. Primary tubules were resuspended in tubule medium consisting of Dulbecco’s modified Eagle medium: F12 culture medium (Life Technologies, Carlsbad, CA, USA), epidermal growth factor (10 ng/ml; Sigma-Aldrich), hydrocortisone (50 nM; Selleck Chemicals, Houston, TX, USA), tri-iodothyronine (32 ng/ml; Selleck), insulin/transferrin/selenium (10 μg/ml/ 5.5 μg/ml/ 5 ng/ml; Life Technologies), and 1% penicillin-streptomycin (Life Technologies). Plates for cell culture were treated with collagen I (Corning, Armonk, NY, USA) 1 hour prior to cell culture.


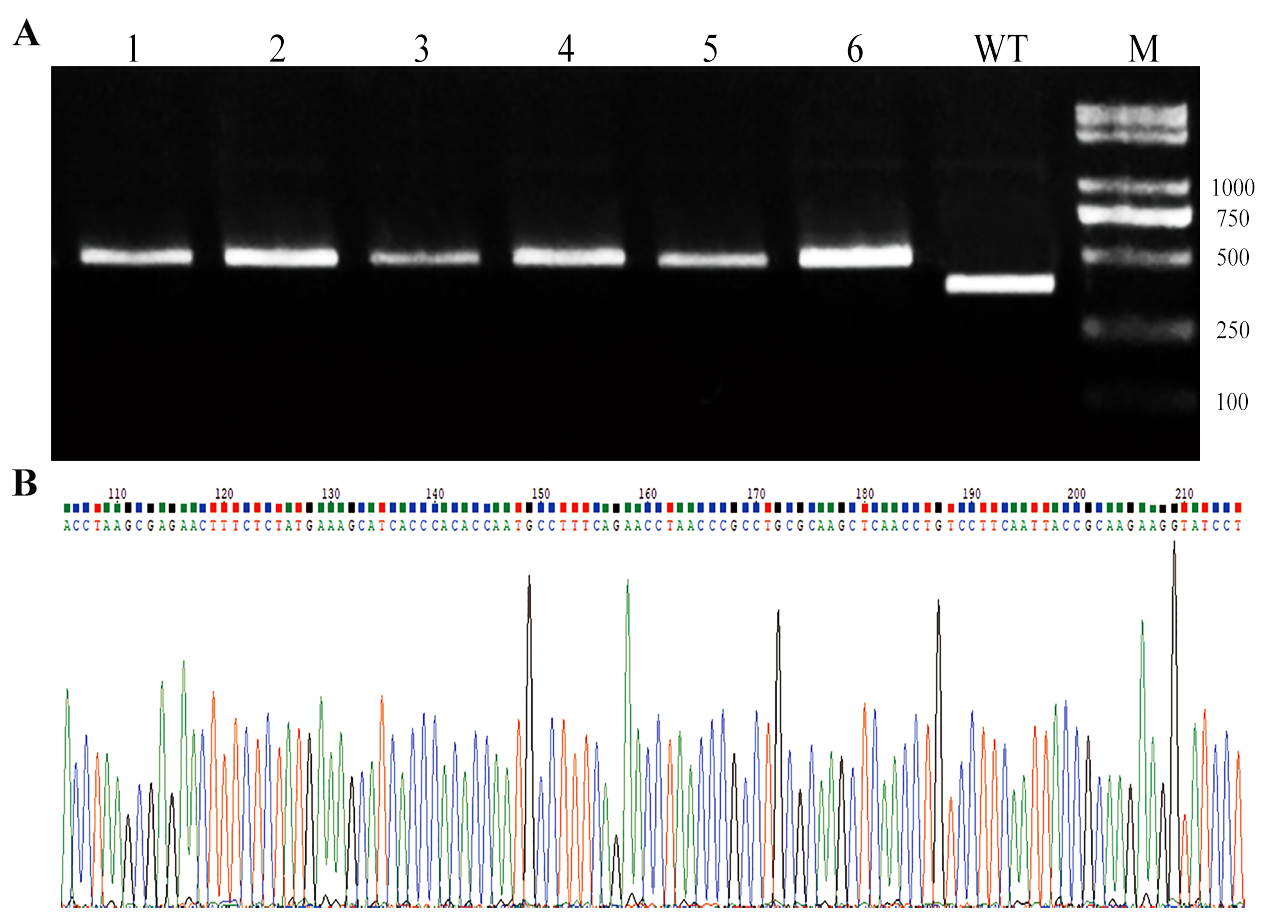


**Figure S1.** Construction and confirmation of TLR-9 knockout mice. (A) Polymerase chain reaction results of WT and KO mice. Lane 1-6: TLR-9 KO mice; lane 7: WT mice, lane 8: Marker. (B) Sequencing results of TLR-9 KO mice. TLR-9: toll-like receptor-9; WT: wild-type; KO: knockout.


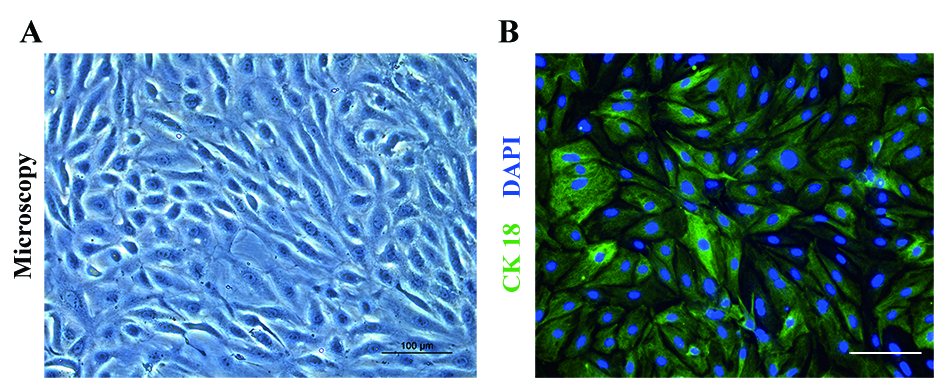


**Figure S2.** Verification of RTECs in vitro. RTECs were isolated and immunofluorescence of CK18 was used to verify the phenotype of epithelial cells. Results were acquired from three independent assays. (A) Light microscopy image of RTECs. (B) Immunofluorescence of CK18. Magnification: 200x. RTECs: renal tubular epithelial cells; CK18: Cytokeratin 18.


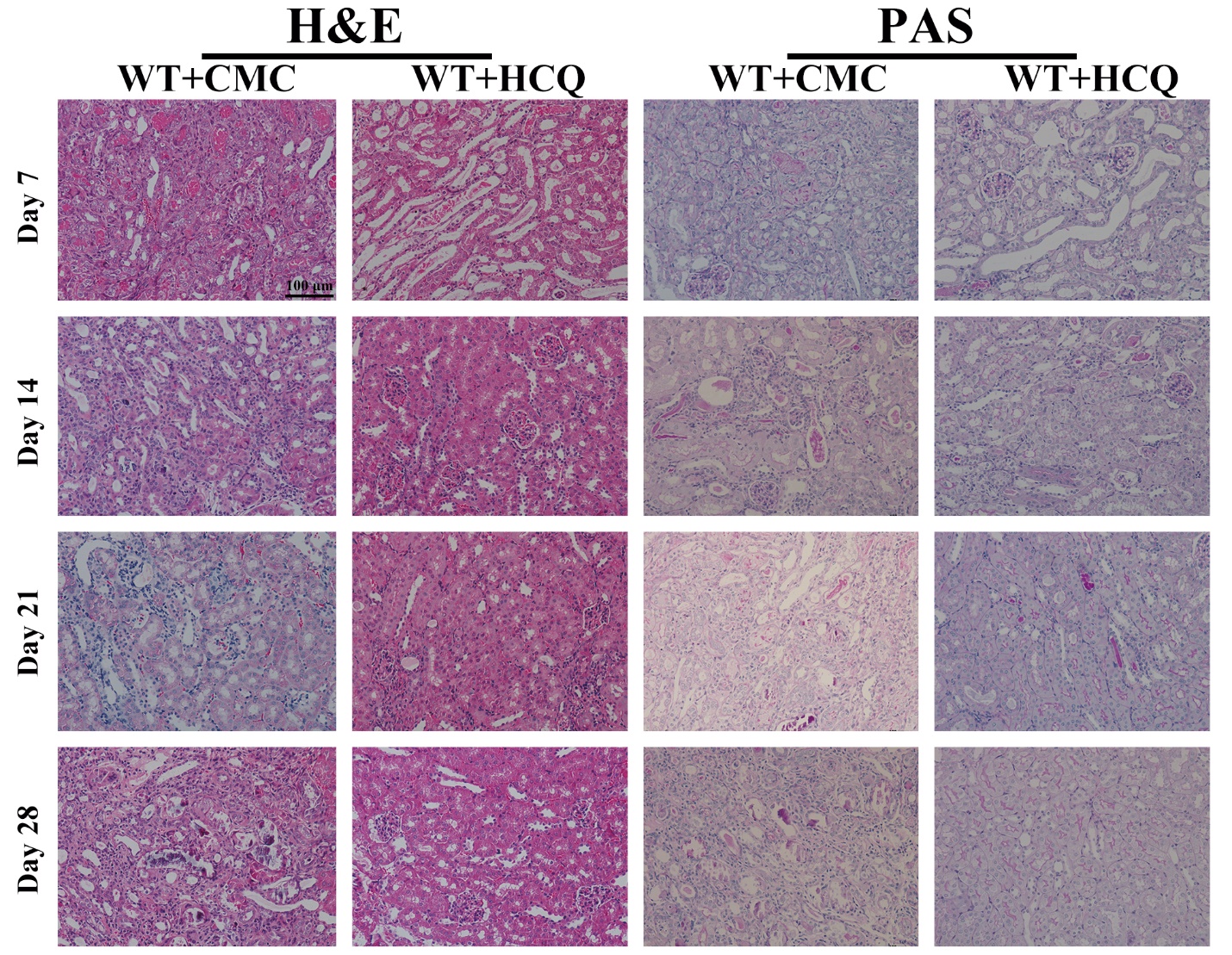


**Figure S3.** HCQ treated accelerates chronic kidney disease recovery following IRI. Mice were exposed to 26 minutes of bIRI and treated with HCQ or CMC. Renal tissues were acquired on day 7, 14, 21, and 28 post-injury. H&E and PAS were used to assess the injury of the renal outer medulla post IRI injury. Tissue sections are representative of five mice per group. Magnification: 200x. NS: no significant difference, WT: wild-type; KO: knockout; H&E: hematoxylin and eosin; PAS: periodic acid–Schiff; CMC: carboxymethyl cellulose; HCQ: hydroxychloroquine.
